# Genetic dissection of quantitative trait loci for flag leaf size in bread wheat (*Triticum aestivum* L.)

**DOI:** 10.3389/fpls.2022.1047899

**Published:** 2022-12-14

**Authors:** Liangen Chen, Zhibin Xu, Xiaoli Fan, Qiang Zhou, Qin Yu, Xiaofeng Liu, Simin Liao, Cheng Jiang, Dian Lin, Fang Ma, Bo Feng, Tao Wang

**Affiliations:** ^1^ Chengdu Institute of Biology, Chinese Academy of Sciences, Chengdu, China; ^2^ University of Chinese Academy of Sciences, Beijing, China; ^3^ The Innovative Academy of Seed Design, Chinese Academy of Sciences, Beijing, China

**Keywords:** flag leaf size, QTL, pleiotropic effect, pyramiding, wheat

## Abstract

Flag leaf size is a crucial trait influencing plant architecture and yield potential in wheat. A recombinant inbred line (RIL) population derived from the cross of W7268 and Chuanyu 12 was employed to identify quantitative trait loci (QTL) controlling flag leaf length (FLL), flag leaf width (FLW), and flag leaf area (FLA) in six environments and the best linear unbiased estimator (BLUE) datasets. Using a 55 K SNP-based genetic map, six major and stable QTL were detected with 6.33–53.12% of explained phenotypic variation. Except for *QFlw.cib-4B.3*, the other five major QTL were co-located within two intervals on chromosomes 2B and 2D, namely *QFll/Fla.cib-2B* and *QFll/Flw/Fla.cib-2D*, respectively. Their interactions and effects on the corresponding traits and yield-related traits were also assessed based on flanking markers. *QFll/Fla.cib-2B* showed pleiotropic effects on spikelet number per spike (SNS). *QFlw.cib-4B.3* and *QFll/Flw/Fla.cib-2D* had effects on grain number per spike (GNS) and thousand-grain weight (TGW). Comparison analysis suggested that *QFll/Fla.cib-2B* was likely a new locus. Two candidate genes, *TraesCS2B03G0222800* and *TraesCS2B03G0230000*, associated with leaf development within the interval of *QFll/Fla.cib-2B* were identified based on expression-pattern analysis, gene annotation, ortholog analysis, and sequence variation. The major QTL and markers reported here provide valuable information for understanding the genetic mechanism underlying flag leaf size as well as breeding utilization in wheat.

## Introduction

Bread wheat (*Triticum aestivum* L.), one of the most widely adapted food crops, provides about a quarter of the calories consumed by humans (Curtis and Halford, 2014). The shape, size, and posture of the leaves together with ear and awn, play a decisive role in the photosynthetic capacity of plants and also regulate many important agronomic traits, such as yield and biotic and abiotic stress responses (Sourdille et al., 2002; Liu et al., 2015).

Flag leaf size, estimated by flag leaf length (FLL), width (FLW), and area (FLA), is an important control of plant structure and is correlated with yield-related traits (Wang et al., 2011; Niu et al., 2022; Zhao et al., 2022). In cereals, flag leaves are the main organ for photosynthesis and plays a crucial role in grain development, such as enhanced proteostasis, lipid remodeling, and nitrogen remobilization (Cohen et al., 2022). Therefore, breeding wheat with optimal leaf morphology has been regarded as an effective method to improve grain yield.

Previously studies attempting to uncover the genetic mechanism of flag leaf morphology in crops showed that flag leaf size was determined by quantitative trait loci (QTL) and significantly influenced by the environment (Coleman et al., 2001; Kobayashi et al., 2003). In rice, the genes that control flag leaf size have been extensively investigated and several major types of signaling pathways have been identified, including the transcription factor signaling, cell expansion pathway, microRNA regulator, and others (Zhang et al., 2009; Tian et al., 2015; Alamin et al., 2017; He et al., 2018; Zhou et al., 2020; Uzair et al., 2021; Huang et al., 2022). In rice, for example, *OsCKX3* mediated the accumulation of cytokinins, the mutant *osckx3* exhibited a larger flag leaf size. (Huang et al., 2022). Map-based cloning in rice identified a GATA family transcription factor was a candidate gene for *SNFL1* and the mutant *snfl1* exhibited a reduction in flag leaf epidermal cell length (He et al., 2018). The *MIR319* gene family contains two members, *Osa-MIR319a* and *Osa-MIR319b*. Overexpression of *MIR319* in rice and *MiR319/TaGAMYB3* module in wheat regulate the number of longitudinal small veins in the leaf, which led to an increase in leaf blade width, and improves grain yield. (Yang et al., 2013; Wang et al., 2014; Jian et al., 2022). *NRL1* encodes the cellulose synthase-like protein D4 (OsCslD4) and plays a critical role in leaf morphogenesis by regulating longitudinal veins and adaxial bulliform cells development (Hu et al., 2010).

The molecular cloning of genes related to flag leaf size in wheat falls behind rice owing to its huge genome. So far, QTL for flag leaf size have been detected on almost all wheat chromosomes based on various genetic populations and environments (Fan et al., 2015; Wu et al., 2016; Liu et al., 2018; Zhao et al., 2018; Jin et al., 2020; Ma et al., 2020; Tu et al., 2021). For example, *TaFLW1*, a major QTL for FLW, was fine-mapped into a 0.2-cM interval on chromosome 5A, which is tightly linked to *Fhb5* (Xue et al., 2013). *QFlw-6A* was fine-mapped to a small interval on chromosome 6A and 10 genes were predicted in this region (Yan et al., 2020). More recently, *QFlw-5B* was narrowed to a 2.5 Mb region and contained 27 predicted genes (Zhao et al., 2022). To date, there is no report of map-based cloning of the gene controlling flag leaf size in wheat.

The present study was undertaken to (i) evaluate the performance of flag leaf size in a recombinant inbred line (RIL) population in multiple environments; (ii) identify QTL for flag leaf size using a wheat 55 K SNP-based genetic map and analyze their effects; (iii) assess relationships between flag leaf size and yield-related traits; (iv) predict candidate genes for major QTL.

## Materials and methods

### Plant materials

A RIL population (WC12, 180 F_9_ lines) derived from the cross of W7268 and Chuanyu 12 (CY12) by the single-seed descend method was used for gene mapping in this study. The wheat line W7268 was selected by our lab. It was characterized by desirable agronomic traits including high SNS, GNS, and flag leaf size. Thus, it has been widely used in wheat breeding programs and several elite varieties have been selected during the past decade. However, the genetic control of flag leaf size in W7268 is uncovered. CY12 is a commercial cultivar with a smaller flag leaf size. In addition, 135 wheat accessions (including 60 modern cultivars and 75 landraces) were used to genotyping. The population was constructed and the accessions were conserved by our laboratory.

### Phenotyping and statistical analysis

RILs of WC12 were evaluated at two experimental sites in three growing seasons: 2018–2019 in Shuangliu (103° 52′ E, 30° 34′ N) (E1); 2018–2019 in Shifang (104°11′ E, 31° 6′ N) (E2); 2019–2020 in Shuangliu (E3); 2019–2020 in Shifang (E4); 2020–2021 in Shuangliu (E5); 2020–2021 in Shifang (E6). A completely randomized block design was used for all of the trials in each environment. Each line was planted in a one-row plot with a row length of 1.2 m, a row spacing of 0.2 m, and 11 seeds per row. Two replicates were employed in each environment. Fertilizer (N: 25%, P_2_O_5_: 10%, K_2_O: 10%) was applied at sowing time at a rate of 450 kg/ha. Field management and disease control were performed in accordance with conventional practices in wheat production.

After anthesis, the main tillers of ten representative plants from each line were selected for measuring flag leaf length (FLL), flag leaf width (FLW), and flag leaf area (FLA). FLL was measured as the distance from the base to the tip of the leaf; FLW was the width of the widest part of the leaf; FLA was derived from the FLL and FLW and estimated as FLL × FLW × 0.75 (Zeuli and Qualset, 1990). Also, the phenotypic values of some yield-related traits were measured. At maturity, ten representative plants were randomly selected to measure agronomic traits, including spikelet number per spike (SNS), spike compactness (SC), grain number per spike (GNS), and fertile tiller number (FTN). Spikelet number per spike (SNS) was determined by counting the number of spikelets in main spikes; SC was calculated by dividing the spike length (SL) by SNS. The main spikes of target plants were then harvested and threshed manually. GNS was then counted manually, and the thousand-grain weight (TGW) and grain yield per plant (GYP) was assessed with SC-G software (Wanshen Detection Technology Co., Ltd., Hangzhou, China).

Basic statistical analysis, frequency distribution analysis, and correlation coefficients analysis among traits were conducted on the phenotypic data using software SPSS25 (Chicago, IL, USA), R 4.1.2, and QTL IciMapping v4.2 (Meng et al., 2015). The best linear unbiased estimator (BLUE) was calculated using the R package “lme4” and used for combined QTL detection, correlation analysis, and effect analysis. Estimation of the broad-sense heritability (*H^2^*) of each trait according to the method described by Smith (Smith et al., 1998). The significance of the difference was measured by the Student’s *t* test (*P* < 0.05), Welch’s two-sample *t* test (*P* < 0.05), and Wilcoxon’s symbol rank-sum (*P* < 0.05) using SPSS25 and R 4.1.2, respectively.

### Linkage map construction and QTL detection

A high-density genetic map containing 2,186 bin markers was constructed using the wheat 55 K SNP array according to a previous study (Liao et al., 2022). This genetic map spanned 2,398.67 cM across 21 chromosomes with an average interval of 1.10 cM/marker. There are 1,598 SNP markers on the map, of which genome A contains 428 markers, genome D contains 467 markers, and genome B contains the most markers (703). In addition, markers derived from three reported genes *Ppd-B1, Rht-B1*, and *Ppd-D1* were integrated into the genetic map for QTL detection (Ellis et al., 2002; Guo et al., 2010; Díaz et al., 2012). They were added to the corresponding chr2B, chr4B, and chr2D genetic map by JoinMap 4.1 (Voorrips et al., 2006).

QTL analysis was conducted using the inclusive composite interval mapping (ICIM) function of QTL IciMapping v4.2 (Meng et al., 2015). Individual environmental QTL values were measured using the bi-parental populations (BIP) module with walking step = 1.00 cM, PIN = 0.001, and LOD score values ≥ 3. QTL with PVE value greater than 20% in at least one environment and could be stably detected in more than four environments (including the BLUE dataset) were considered as the major ones. QTL with common flanking markers or less than 1 cM apart were considered identical. QTL were named according to the International Rules of Genetic Nomenclature (https://wheat.pw.usda.gov/ggpages/wgc/98/Intro.htm). The “cib” represents “Chengdu Institute of Biology”.

### Marker development and genotyping

To develop new markers within the mapped interval, variants between parental lines were detected using exome sequencing. Exon capture, sequencing, and analysis were performed by Bioacme Biotechnology Co., Ltd (Wuhan, China, http://www.whbioacme.com).

On the basis of the preliminary QTL mapping result, new Kompetitive Allele Specific PCR (KASP) markers were developed within the interval of major QTL. According to the exome sequencing result, the variant sites (including SNPs and InDels) between W7268 and CY12 were screened. Only SNPs with differences between W7268 and CY12 at the major QTL, by referring to the Chinese Spring (CS) reference genome IWGSC RefSeq v2.1 (http://www.wheatgenome.org), were converted into KASP markers. KASP assay reaction procedure and data analysis implemented according to the method depicted by Ji (Ji et al., 2020).

### Conditional QTL analysis for FLA

Conditional QTL analysis is an excellent tool for interpreting the relationship between complex traits and components at the QTL level (Cui et al., 2011). We carried out conditional QTL analysis as described by Liu (Liu et al., 2018). QTL IciMapping v4.2 (Meng et al., 2015) was used to identify the conditional loci with conditional phenotypic values. The conditional phenotypic values (T1|T2), which means the value of Trait 1 conditional on Trait 2, were obtained using QGAStation2.0 (Chen et al., 2012). Here, ‘FLA|FLL’ and ‘FLA|FLW’ refer to the value of FLA excluding the influences of FLL and FLW, respectively. In the analysis of QTL mapping results, if a QTL was detected only in unconditional QTL analysis, the locus was considered to have a large contribution to the corresponding trait; whereas a QTL was considered to be unassociated with the corresponding trait if it was detected in both conditional and unconditional QTL analysis.

### Prediction of candidate gene

Physical intervals of the major QTL detected in this study were obtained by blasting against (E-value of 1e-5) their flanking markers sequences to genome sequences of CS reference genome IWGSC RefSeq v2.1 (http://www.wheatgenome.org). The annotations and functions of genes between flanking markers were further analyzed using UniProt (https://www.uniprot.org/). The expression pattern analysis of candidate genes was performed using Gene Expression of Triticeae Multi-omics Center (http://202.194.139.32/expression/wheat.html) (Ma et al., 2021) and Wheat Expression Browser (http://www.wheat-expression.com) (Ramírez-González et al., 2018). The circle graph of expression values was drawn using the ggplot2 package in R 4.1.2. Analysis of orthologous genes in wheat and rice was carried out using the Triticeae-Gene Tribe (http://wheat.cau.edu.cn/TGT/) (Chen et al., 2020). Furthermore, to analyze the potential candidate genes, SNPs in the target regions were collected using the exome sequencing result.

## Results

### Phenotypic variation of flag leaf-related traits

**Figure 1:**
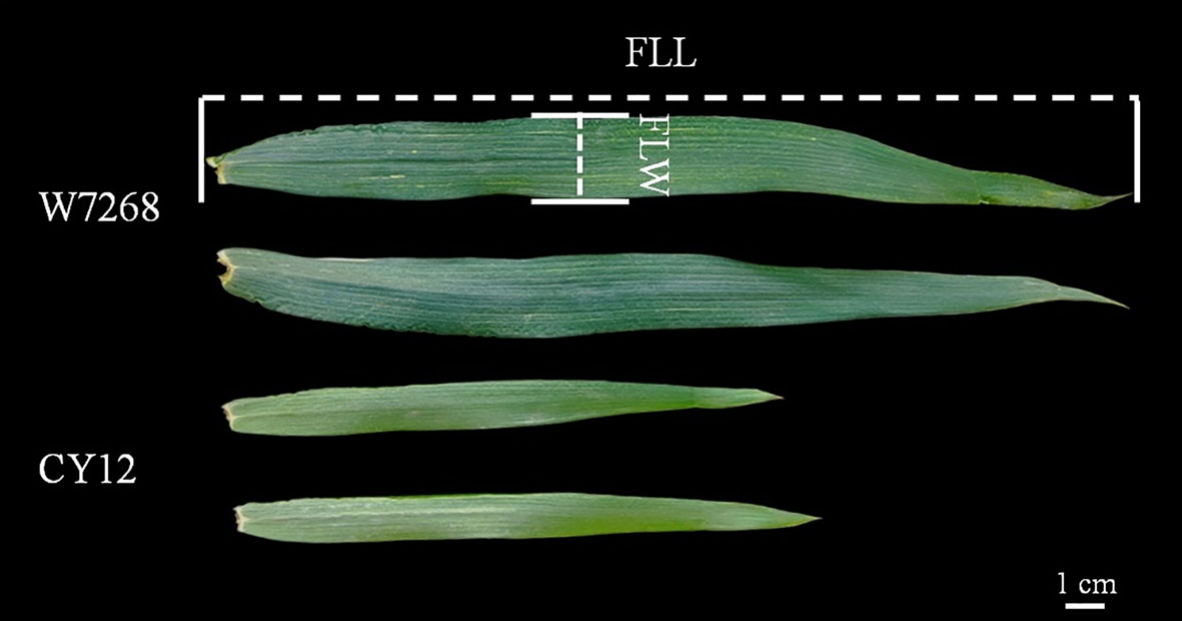
Morphology of the flag leaf size of W7268 and Chuanyu 12 at flowering stage from Shuangliu 2020-2021 trail.

**Figure 2:**
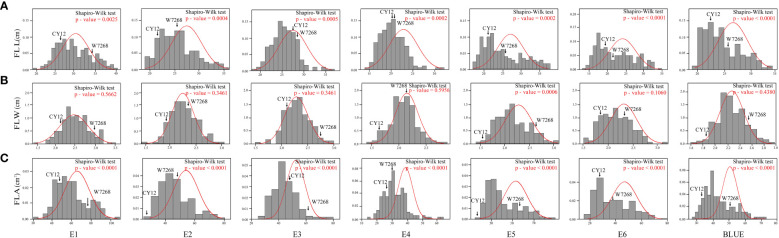
Frequency distribution of the WC12 lines for flag leaf length (FLL) **(A)**, flag leaf width (FLW) **(B)**, and flag leaf area (FLA) **(C)** in six environments and the BLUE datasets.

The phenotypic means of flag leaf-related traits (including FLL, FLW and FLA) for the parents and the population were listed in **Table 1**, as well as basic statistics from plants grown in six environments and the BLUE datasets. Significant differences in FLL, FLW, and FLA between W7268 and CY12 were observed (**Figure 1** and **Table 1**). W7268 had significantly higher values for FLL (except E3 and E4), FLW, and FLA than those of CY12. The flag leaf-related traits of the WC12 population ranged from 19.50 to 33.80 cm for FLL, 1.62 to 2.95 cm for FLW, and 27.80 to 73.20 cm^2^ for FLA, in BLUE datasets, respectively. The estimated *H^2^* for FLL, FLW, and FLA were 0.88, 0.92, and 0.89, respectively. These results indicated that FLL, FLW, and FLA were environmentally stable and were mainly determined by genetic factors. A pattern of continuous distributions for FLL, FLW, and FLA was observed in each environment and the BLUE dataset of the WC12 population, suggesting that they were common quantitative traits and controlled by multi-genes (**Figure 2**). Furthermore, significant and positive correlations among FLL, FLW, and FLA were detected among six environments, with Pearson’s correlations (*r*) of 0.21–0.93 in the WC12 population (**Supplementary Figure 1**).

**Table 1:** Phenotypic performance and distribution of flag leaf length (FLL), flag leaf width (FLW) and flag leaf area (FLA) in parents and WC12 lines in different environments.

Trait	Environment	Parents		The WC12 lines				*H^2^*
		W7268	CY12	Range	Mean ± SD	Skeness	Kurtosis	
FLL (cm)	E1	34.00	27.17***	19.96–41.22	30.09 ± 4.67	0.34	-0.68	0.88
	E2	25.35	22.55*	19.08–35.90	25.45 ± 3.56	0.61	0.02
	E3	28.44	27.25	18.53–36.38	25.52 ± 2.96	0.68	1.46
	E4	19.32	19.11	14.01–31.57	20.25 ± 2.78	0.76	1.38
	E5	26.29	21.20*	16.15–37.33	24.52 ± 5.68	0.65	-0.75
	E6	22.49	19.26*	13.58–34.78	22.14 ± 5.05	0.46	-0.9
	BLUE	25.90	22.50	19.50–33.80	24.59 ± 3.34	0.68	-0.44	
FLW (cm)	E1	3.03	2.23****	1.65–3.52	2.63 ± 0.33	0.12	-0.16	0.92
	E2	2.45	2.11***	1.68–3.02	2.38 ± 0.23	0.23	-0.07
	E3	2.84	2.19****	1.58–3.00	2.31 ± 0.24	0.16	0.38
	E4	2.12	1.64****	1.46–2.84	2.09 ± 0.24	0.11	0.37
	E5	2.60	1.55****	1.48–3.09	2.16 ± 0.32	0.46	-0.2
	E6	2.34	1.80****	1.53–3.11	2.16 ± 0.30	0.26	-0.35
	BLUE	2.56	1.91	1.62–2.95	2.28 ± 0.24	0.26	0.12	
FLA (cm^2^)	E1	77.44	45.78****	23.93–107.48	59.94 ± 15.84	0.54	-0.34	0.89
	E2	46.73	36.04**	29.12–78.33	45.82 ± 10.04	0.86	0.49
	E3	60.65	42.38***	26.37–78.76	44.78 ± 8.72	0.97	2.06
	E4	30.72	23.61***	15.38–62.65	32.13 ± 7.50	1.01	2.38
	E5	51.47	24.66****	19.48–86.69	40.77 ± 14.98	0.92	-0.09
	E6	39.35	26.28****	17.35–75.78	37.00 ± 13.09	0.648	-0.564
	BLUE	50.60	32.90	27.80–73.30	43.26 ± 9.47	0.828	0.121	

BLUE best linear unbiased estimator; *H^2^* broad-sense heritability; SD standard deviation; *, **, ***, and **** represent significant at *P* < 0.05, *P* < 0.01, *P* < 0.005, and *P* < 0.001, respectively.

### Correlation analysis between flag leaf-related and yield-related traits

**Figure 3:**
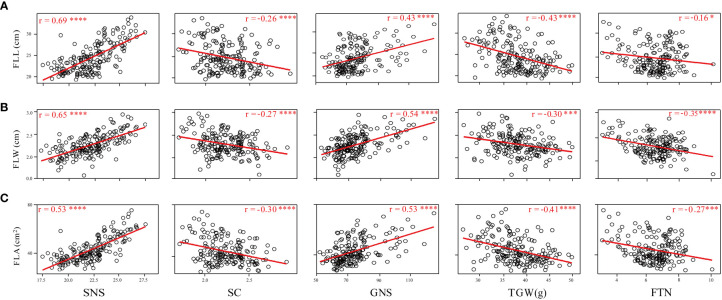
Coefficients of pairwise Pearson’s correlations between flag leaf length **(A)**, flag leaf width **(B)**, and flag leaf area **(C)** and yield-related traits in the WC12 population. *, *** and **** represent significant at *P* < 0.05, *P* < 0.005 and *P* < 0.001, respectively.

Phenotypic correlations between FLL, FLW, and FLA were assessed using the BLUE datasets. FLL was significantly and positively correlated with FLW and FLA (*P* < 0.001); FLW was also significantly and positively correlated with FLA (*P* < 0.001) (**Supplementary Table 1**). The relationship between flag leaf-related traits and SNS, SC, GNS, TGW, and FTN was also assessed (**Figure 3**). The results showed that FLL, FLW, and FLA were significantly and positively correlated with SNS and GNS (*P* < 0.001), and significantly and negatively associated with SC (*P* < 0.001) and TGW (*P* < 0.001). Significant and negative correlations were detected between FLL and FTN (*P* < 0.05), FLW and FTN (*P* < 0.001), and FLA and FTN (*P* < 0.005).

### QTL detection for flag leaf-related traits

A total of 48 QTL for FLL, FLW, and FLA were detected in the WC12 population and located on chromosomes 1A, 2A, 5A, 7A, 1B, 2B, 3B, 4B, 5B, 6B, 7B, 2D, 4D, 5D and 7D, respectively (**Supplementary Table 2**). Among them, six major QTL located on chromosomes 2B, 4B, and 2D could be consistently identified in more than three environments and the BLUE dataset, and thus, they were considered to be environmentally stable (**Table 2**).

**Table 2:** Major and stable quantitative trait loci (QTL) for flag leaf length (FLL), flag leaf width (FLW), and flag leaf area (FLA) identified across multiple environments in the WC12 population.

Trait	QTL	Environment	Interval (cM)	Flanking markers	LOD	PVE (%)	Add
FLL	*QFll.cib-2B.2*	E1	28.5–30.5	AX-109305292–KA12	7.38	17.99	-1.93
		E2	28.5–30.5	AX-109305292–KA12	4.89	13.15	-1.16
		E4	28.5–30.5	AX-109305292–KA12	3.70	7.31	-0.76
		E5	28.5–30.5	KA12–KA15	13.88	30.87	-3.14
		E6	29.5–30.5	KA12–KA15	5.91	14.20	-1.89
		BLUE	28.5–30.5	AX-109305292–KA12	10.61	24.92	-1.63
	*QFll.cib-2D*	E1	124.5–128.5	AX-111956072–Ppd-D1	23.04	45.03	3.17
		E2	127.5–128.5	AX-111956072–Ppd-D1	17.42	36.65	2.16
		E4	117.5–128.5	AX-110289516–AX-111956072	3.78	6.93	0.75
		E5	119.5–127.5	AX-110289516–AX-111956072	15.01	31.35	3.59
		E6	123.5–128.5	AX-110289516–AX-111956072	29.48	53.12	3.77
		BLUE	121.5–128.5	AX-110289516–AX-111956072	20.08	40.02	2.23
FLW	*QFlw.cib-4B.3*	E1	90.5–92.5	AX-110928817–Rht-B1	6.90	20.57	0.13
		E2	88.5–90.5	AX-111585045–AX-110928817	8.18	19.60	0.10
		E4	90.5–92.5	Rht-B1–AX-111497396	6.83	16.42	0.10
		E5	88.5–90.5	AX-111735154–AX-111585045	8.71	20.31	0.15
		BLUE	90.5–92.5	AX-110928817–Rht-B1	6.78	20.74	0.09
	*QFlw.cib-2D.1*	E1	119.5–128.5	AX-110289516–AX-111956072	11.79	26.43	0.20
		E2	127.5–129.5	AX-111956072–Ppd-D1	4.61	11.30	0.08
		E5	120.5–128.5	AX-110289516–AX-111956072	10.05	22.87	0.16
		E6	127.5–129.5	AX-111956072–Ppd-D1	19.91	40.43	0.19
		BLUE	119.5–128.5	AX-110289516–AX-111956072	9.89	22.26	0.12
FLA	*QFla.cib-2B.2*	E1	29.5–30.5	KA12–KA15	6.66	15.47	-6.32
		E2	27.5–29.5	AX-109305292–KA12	4.71	12.47	-3.20
		E3	30.5–33.5	KA13–KA17	3.51	6.33	-2.19
		E5	28.5–30.5	KA12–KA15	11.27	25.33	-7.58
		BLUE	29.5–30.5	KA12–KA15	9.31	21.38	-4.38
	*QFla.cib-2D*	E1	122. –128.5	AX-110289516–AX-111956072	17.99	37.14	9.90
		E2	127.5–128.5	AX-111956072–Ppd-D1	12.88	28.60	5.37
		E5	120.5–128.5	AX-110289516–AX-111956072	14.61	31.39	8.75
		BLUE	122.5–128.5	AX-110289516–AX-111956072	18.05	37.19	5.88

PVE phenotypic variation explained; LOD logarithm of the odd; Add additive effects (positive values indicate that alleles from W7268 are increasing the trait scores, and negative values indicate that alleles from CY12 are increasing the trait scores); BLUE best linear unbiased estimator.

**Figure 4:**
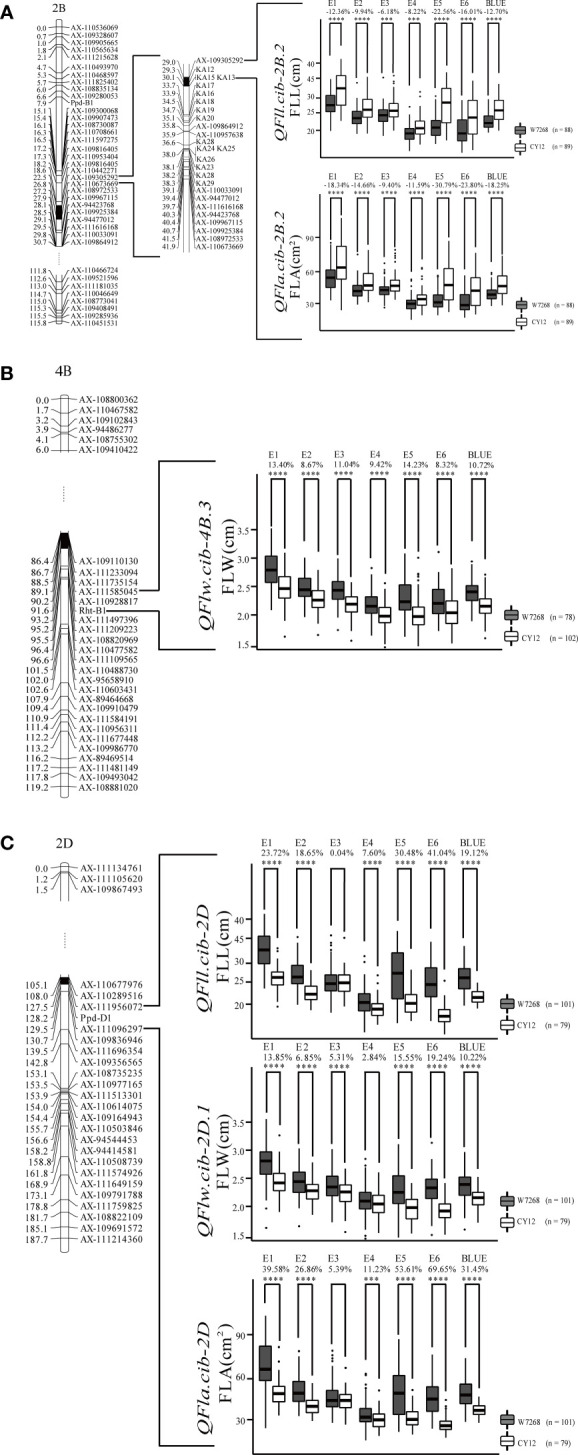
Genetic map of major quantitative trait loci (QTL), *QFll.cib-2B.2* and *QFla.cib.2B.2* **(A)**, *QFlw.cib-4B.3* **(B)**, *QFll.cib-2D, QFlw.cib-2D.1*, and *QFla.cib.2D* **(C)**, and their effects on corresponding traits in WC12 population. W7268 and CY12 represent lines with alleles from W7268 and CY12, respectively; ***, and **** represent significant at *P* < 0.005, and *P* < 0.001, respectively.

Two major QTL for FLL, *QFll.cib-2B.2* and *QFll.cib-2D*, were detected. *QFll.cib-2B.2* was identified in five environments and the BLUE dataset. It explained 7.31–30.87% of the phenotypic variance with the LOD values ranging from 3.70 to 13.88. *QFll.cib-2D* was identified in five environments and the BLUE dataset with the LOD values of 3.78–29.48, explaining 6.93–53.12% of the phenotypic variance. The positive alleles of *QFll.cib-2B.2* and *QFll.cib-2D* were contributed by CY12 and W7268, respectively (**Figure 4** and **Table 2**). Two minor QTL *QFll.cib-1B* and *QFll.cib-5D.1* were detected in three environments, explaining 2.25–3.38%, and 3.73–5.09%, respectively, of phenotypic variation (**Supplementary Table 2**).

Two major QTL and six minor QTL for FLW were detected on chromosomes 2B, 3B, 4B, 2D, 5D, and 7D. The two major QTL, *QFlw.cib-4B.3* and *QFlw.cib-2D.1*, were identified in four environments and the BLUE datasets. *QFlw.cib-4B.3* explained 16.42–20.74% of the phenotypic variance with the LOD values of 6.78–8.71. *QFlw.cib-2D.1* explained 11.30–40.43% of the phenotypic variance with the LOD values of 4.61–19.91. The positive alleles of these two loci were contributed by W7268 (**Figure 4** and **Table 2**). Minor QTL, *QFlw.cib-3B.2*, *QFlw.cib-4B.1*, *QFlw.cib-7D.1*, and *QFlw.cib-7D.2*, were detected in less than four environments, explaining 2.30–8.90%, 9.40–12.17%, 2.95–6.38%, 2.84–5.21%, respectively, of phenotypic variation. *QFlw.cib-2B.1* and *QFlw.cib-5D.1* were identified in four environments and the BLUE datasets, explaining 8.57–13.52% and 4.08–7.58% of the phenotypic variance, respectively (**Supplementary Table 2**).

Two major QTL associated with FLA were detected. *QFla.cib-2B.2* was detected in four environments and the BLUE dataset with LOD values ranging from 3.51 to 11.27. It explained 6.33–25.33% of the phenotypic variance. *QFla.cib-2D*, detected in three environments and the BLUE dataset, had LOD values of 12.88–18.05 and accounted for 28.6–37.19% of the phenotypic variance. The positive alleles of *QFla.cib-2B.2* and *QFla.cib-2D* were contributed by CY12 and W7268, respectively (**Figure 4** and **Table 2**).

One minor QTL *QFlw.cib-2B.1* and two major QTL, *QFll.cib-2B.2*and *QFla.cib-2B.2*, shared the same flanking marker *KA12* on chromosome 2B (**Figure 4A** and **Supplementary** **Table 2**). Meanwhile, three QTL *QFll.cib-2D*, *QFlw.cib-2D.1*, and *QFla.cib-2D* were co-localized in the interval of *AX-110289516–AX-111956072* (**Figure 4C** and **Table 2**). Thus, the two loci were temporarily designated as *QFll/Fla.cib-2B* and *QFll/Flw/Fla.cib-2D.*

According to the interval of *QFll/Fla.cib-2B*, KASP markers (*KA01* to *KA29*) were developed and integrated into the genetic map based on the exome sequencing result of the two parents (**Supplementary Table 3**). Among them, four markers, *KA12*, *KA13*, *KA15*, and *KA17*, were found to be closely linked to *QFll/Fla.cib-2B* (**Figure 4A**).

### Conditional QTL analysis for FLA

FLA is a complex trait composed of FLL and FLW. We performed conditional QTL analysis of FLA in the WC12 population to further evaluate the effect of FLL and FLW on FLA. The results showed that when FLA was conditional on FLL, the LOD values of *QFla.cib-2B.2* and *QFla.cib-2D* were significantly reduced; when FLW was conditioned, the LOD values were lower than those in the unconditional analysis but remained at a high level (**Supplementary Figure 2**). These results indicated that FLL was primarily responsible for FLA in the WC12 population.

### Effects of major QTL on corresponding traits

In the WC12 population, we identified seven major QTL (**Table 2**). Their effects on corresponding traits were assessed on the basis of flanking markers. As expected, lines in the WC12 population with the positive alleles at the three loci showed significantly higher values of the corresponding traits in all environments and the combined data than those with the negative alleles (except for E3 of *QFll.cib-2D*, E4 of *QFlw.cib-2D.1*, and E3 of *QFla.cib-2D*) (**Figure 4**).

### Effects of major QTL on yield-related traits in the mapping population

**Figure 5:**
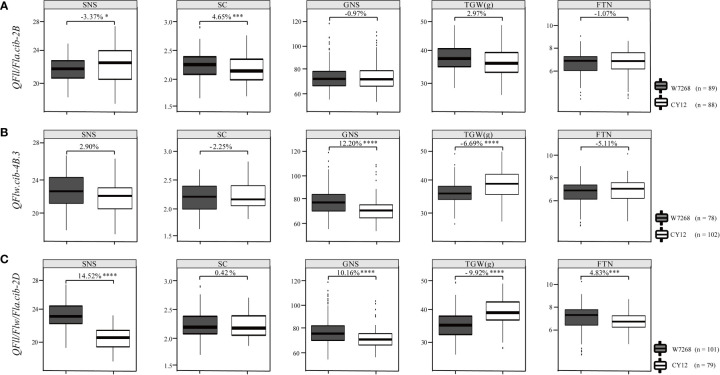
Effects of *QFll/Fla.cib-2B* **(A)**, *QFlw.cib-4B.3* **(B)**, and *QFll/Flw/Fla.cib-2D* **(C)** on spikelet number per spike (SNS), spikelet compactness (SC), grain number per spike (GNS), thousand grain weight (TGW), and fertile tiller number (FTN) in WC12 population. W7268 and CY12 represent lines with alleles from W7268 and CY12, respectively; *, ***, and **** represent significant at *P* < 0.05, *P* < 0.005, and *P* < 0.001, respectively.

We further evaluated the effects of major QTL on yield-related traits using the BLUE datasets. Compared with lines harboring the alleles from CY12 at *QFll/Fla.cib-2B*, lines with the alleles from W7268 had lower SNS (*P* < 0.05), but higher SC (*P* < 0.005). *QFll/Fla.cib-2B* had no significant effects on GNS, TGW, and FTN (**Figure 5A**). For *QFlw.cib-4B.3*, lines containing the alleles from W7268 had higher GNS (*P* < 0.001) but lower TGW (*P* < 0.001) than lines containing the alleles from CY12 (**Figure 5B**). No significant difference in SNS, SC, and FTN was observed between the two groups. For *QFll/Flw/Fla.cib-2D*, lines possessing the alleles from W7268 had significantly higher SNS (*P* < 0.001), GNS (*P* < 0.001), and FTN (*P* < 0.005) than lines containing the alleles from CY12, but had significantly lower TGW (*P* < 0.001) (**Figure 5C**).

### Analyses of additive effects of major QTL for FLL, FLW, and FLA

Since multiple QTL for FLL (*QFll.cib-2B.2* and *QFll.cib-2D*), FLW (*QFlw.cib-4B.3* and *QFlw.cib-2D.1*), and FLA (*QFla.cib-2B.2* and *QFla.cib-2D*) could be simultaneously detected, we further analyzed their additive effects on the corresponding traits.

For FLL, lines with the combination of positive alleles of *QFll.cib-2B.2* and *QFll.cib-2D* significantly (*P* < 0.001) increased FLL by 31.68% compared with those without any positive alleles. Either lines with a single positive allele from *QFll.cib-2B.2* or *QFll.cib-2D* also significantly (*P* < 0.001) increased FLL by 5.98% and 11.43%, respectively, compared with those possessing no positive alleles of FLL QTL. These results indicated that the combination of *QFll.cib-2B.2* and *QFll.cib-2D* had the largest effect, followed by *QFll.cib-2D* and *QFll.cib-2B.2*, respectively (**Supplementary Figure 3**).

For FLW, compared with lines without any positive alleles, lines with the combination of positive alleles of *QFlw.cib-4B.3* and *QFlw.cib-2D.1*, lines harboring the positive allele from *QFlw.cib-4B.3* and lines with the positive allele from *QFlw.cib-2D.1*, all significantly (*P* < 0.001) increased FLW by 20.87%, 8.21%, and 8.40%, respectively. These results showed that the combination of *QFlw.cib-4B.3* and *QFlw.cib-2D.1* had the largest effect, followed by the single allele *QFlw.cib-4B.3*, and *QFlw.cib-2D.1*, respectively (**Supplementary Figure 3**).

For FLA, lines with the combination of positive alleles of *QFla.cib-2B.2* and *QFla.cib-2D*, lines possessing the positive allele from *QFla.cib-2D*, and lines with the positive allele from *QFla.cib-2B.2*, all significantly (*P* < 0.001) increased FLA than lines without any positive alleles by 52.14%, 20.08%, and 9.89%, respectively. These results suggested that the combination of both *QFla.cib-2B.2* and *QFla.cib-2D* had the largest effect, followed by *QFla.cib-2D* and *QFla.cib-2B.2*, respectively (**Supplementary Figure 3**).

### Potential candidate genes for *QFll/Fla.cib-2B*

According to the CS reference genome (IWGSC RefSeq v2.1), there were 56 annotated high-confidence genes in the interval of *QFll/Fla.cib-2B* (**Supplementary Table 4**). Expression pattern analysis showed that 36 (Triticeae Multi-omics Center) and 23 (Wheat Expression Browser) genes were expressed in the leaf, respectively (**Supplementary Figure 4**). Gene annotation and orthologous gene analyses (**Supplementary Table 4**), combined with previous expression pattern analysis, suggested that *TraesCS2B03G0222800* and *TraesCS2B03G0230000* were likely to be associated with flag leaf development and growth. Six SNPs, two located in introns and four in the coding region, were detected in *TraesCS2B03G0230000* between W7268 and CY12 by sequence analysis. Among the four SNPs in the exons, there were two synonymous SNPs and two non-synonymous SNPs (Arg to Trp, and Ala to Val) (**Supplementary Table 5**).

## Discussion

### Comparison of the major QTL to those reported previously

In this study, seven major and stable QTL were identified in multiple environments, explaining 6.33–53.12% of phenotypic variations. These QTL will benefit cloning and marker-assisted selection (MAS) in wheat breeding.

We compared their physical positions to those reported in previous studies. The physical position of *QFlw.cib-4B.3*, located at 28.6–43.54 Mb on chromosome 4B, overlapped with the previously reported *Rht-B1* (33.61–33.62 Mb on chromosome 4B). Meanwhile, one of the flanking makers of *QFlw.cib-4B.3* is *Rht-B1* (**Supplementary Figure 5**). *QFll/Flw/Fla.cib-2D* comprising of *QFll.cib-2D*, *QFlw.cib-2D.1*, and *QFla.cib-2D* were located at 36.20-59.78 Mb on chromosome 2D and overlapped to *Ppd-D1* (36.20–36.21 Mb on chromosome 2D). Meanwhile, according to the genetic map, one of the flanking makers of *QFll/Flw/Fla.cib-2D* is *Ppd-D1* (**Supplementary Figure 6**). In addition, it has been reported that *Rht-B1* (Wen et al., 2013; Tang et al., 2018; Jobson et al., 2019) and *Ppd-D1* (Liu et al., 2001) could significantly affect the length, width, and area of flag leaf in different genetic backgrounds and environments, which is consistent with this study. These results suggested that *Rht-B1* and *Ppd-D1* were likely the candidates of *QFlw.cib-4B.3* and *QFll/Flw/Fla.cib-2D*, respectively.

*QFll.cib-2B.2* and *QFla.cib-2B.2* designated as *QFll/Fla.cib-2B* together with *QFlw.cib-2B.1*, were co-located between 62.03 Mb and 68.71 Mb on chromosome 2B. Two reported QTL, close to the interval of *QFll/Fla.cib-2B*, *QFLL-2B* and *QFLA-2B* (Liu et al., 2018), were co-located in 54.44–66.50 Mb on chromosome 2B (**Supplementary Figure 7**), showing a significant effect on FLL and FLA, respectively, but not on FLW. In addition, these two loci were not stable (only detected in two environments), and *QFLA-2B* was a minor QTL. In the present study, we detected *QFll/Fla.cib-2B* and *QFlw.cib-2B.1* in five environments (including the BLUE datasets) and the locus had an effect on FLW. These results indicate that *QFll/Fla.cib-2B* may be different from *QFLL-2B* and *QFLA-2B.*

Because *Ppd-B1* has a similar physical interval (63.36–63.37 Mb on chromosome 2B) to *QFll/Fla.cib-2B*, we used the functional marker of *Ppd-B1* to perform the comparison. By genotyping the WC12 lines, we integrated it into the genetic map and the result showed that *Ppd-B1* was genetically separate from *QFll/Fla.cib-2B* (**Figure 4A**). As a result, *QFll/Fla.cib-2B* is likely a novel locus.

### Relationships between flag leaf size and yield-related traits and pleiotropic effects of major QTL

Optimizing flag leaf size, including length, width, and area, plays an important role in increasing grain yield (Zhao et al., 2018). In wheat, the flag leaf size was significantly correlated with yield-related traits (Cui et al., 2003; Wang et al., 2011; Fan et al., 2015; Liu et al., 2018; Zhao et al., 2022). In the present study, we also found that FLL, FLW, and FLA showed a positive correlation to SNS and GNS, and a negative correlation to SC, FTN, and TGW (**Figure 3**). This result suggested that larger flag leaf benefits from forming more SNS and GNS but hinders the tillering and grain weight increase.

W7268 is an elite line characterized by high SNS and GNS in the Yangzi River region of China. It has been widely used in wheat breeding programs and several varieties have been selected using W7268 as one of the parents. In the present study, we found that *QFlw.cib-4B.3* and *QFll/Flw/Fla.cib-2D* have various pleiotropic effects on SNS and GNS, which may contribute to the higher yield of W7268. Moreover, *QFll/Fla.cib-2B* from CY12 also had additive effects on SNS and GNS and may be an unreported locus with potential to increase yield (**Figure 5**). Thus, cloning and utilization of these QTL will be valuable for grain yield improvement by optimizing flag leaf size.

To analyze the utilization of the positive alleles of the major QTL during artificial selection, we used their flanking markers to genotype 135 wheat accessions (75 of landraces and 60 of cultivars), and the results were shown in **Supplementary Table 6.** The number of landraces with the positive alleles of *QFll/Fla.cib-2B*, *QFlw.cib-4B.3*, *QFll/Flw/Fla.cib-2D* was 21, 36 and 47, or 28.0%, 46.2% and 62.7%, respectively. At the same time, the number of accessions with positive alleles in cultivars was 30, 22 and 8, or 50.0%, 36.7% and 13.3%, respectively. This result suggested that the positive alleles of *QFlw.cib-4B.3*, *QFll/Flw/Fla.cib-2D* were not preferred by breeders and their distribution was decreased, while *QFll/Fla.cib-2B* was enriched during selection.

Based on our results, *QFlw.cib-4B.3* and *QFll/Flw/Fla.cib-2D* may be allelic to *Rht-B1* and *Ppd-D1*, respectively. And the positive allele for *QFlw.cib-4B.3* may be *Rht-B1b* and for *QFll/Flw/Fla.cib-2D* may be *Ppd-D1b*. According to previous reports (Yang et al., 2009; Guo et al., 2010; Tang et al., 2012; Wang et al., 2013; Xu et al., 2014; Bai et al., 2015; Zhang et al., 2016), the distribution of *Rht-B1a* and *Ppd-D1a* was enriched during the breeding process compared to *Rht-B1b* and *Ppd-D1b* in China, which was consistent with our study. The dwarf gene *Rht-B1b* could reduce the thousand-grain weight, which may be the reason why breeders did not prefer this genotype (Xu et al., 2014; Zhang et al., 2016). *Ppd-D1a*, insensitive to photoperiod, facilitated flowering and allowed wheat to finish filling before the onset of summer heat. (Yang et al., 2009; Guo et al., 2010; Bai et al., 2015).

As shown in **Supplementary Figure 8**, significant differences in GYP were identified between lines with positive and negative alleles, with one, three, and four environments for *QFlw.cib-4B.3*, *QFll/Fla.cib-2B*, and *QFll/Flw/Fla.cib-2D*, respectively. As expected, lines with the negative alleles of *QFlw.cib-4B.3* and *QFll/Flw/Fla.cib-2D* showed higher GYP values, consistent with their distribution changes during the artificial selection. For *QFll/Fla.cib-2B*, lines with the negative allele had a higher GYP than the positive allele.

The significant effects of these flag leaf-related QTL on different traits, their distribution in different accessions, and various impacts on yield, suggested that utilization of them will help to improve grain yield by optimizing flag leaf size.

### Potential candidate genes for *QFll/Fla.cib-2B*

In the physical interval of *QFll/Fla.cib-2B* in the CS genome, 36 (Triticeae Multi-omics Center) and 23 (Wheat Expression Browser) genes expressed in leaves were screened (**Supplementary Figure 4** and **Supplementary Table 4**). According to expression-pattern analysis, gene annotation, and ortholog analysis, two candidate genes *TraesCS2B03G0222800* and *TraesCS2B03G0230000* were preliminarily selected to be associated with flag leaf development. Among them, *TraesCS2B03G0222800* encodes a pseudo-response regulator and is an ortholog of rice *Os07g0695100*, which is also known as *DTH7 (Days to heading 7)/Ghd7.1(Grain number, plant height, and heading date 7)*, a pleiotropic gene controlling tassel stage, plant height and the number of glumes per spike in rice (Yan et al., 2013). A recent study has shown that *Ghd7.1* mutations resulted in reduced leaf size in rice, and allelic variance analysis has verified that *Ghd7.1* is a functional candidate gene for leaf size (Tang et al., 2018). *TraesCS2B03G0230000* encodes an omega-3 fatty acid desaturase, which is associated with the production of alpha-linolenic acid, a major class of fatty acids found in the membrane lipid cells of higher plants. (John et al., 1995). Plastid omega-3 desaturase activity is necessary to increase the level of alpha-linolenic acid in extra plastid lipids during leaf development (Horiguchi et al., 1998). Sequence analysis also revealed abundant sequence polymorphisms in *TraesCS2B03G0230000* between W7268 and CY12 (**Supplementary Table 5**). Therefore, *TraesCS2B03G0222800* and *TraesCS2B03G0230000* may be potential candidates for map-based cloning in the future.

## Data availability statement

The original contributions presented in the study are included in the article/**Supplementary Material**. Further inquiries can be directed to the corresponding authors.

## Author contributions

LC carried out the entire study and wrote the draft manuscript. ZX constructed the mapping population. XF and QZ assisted in field trials. QY, SL, XL, CJ, DL, and FM participated in phenotyping and data analysis. TW guided the entire study and discussed the results. BF designed the experiments, participated in data analysis, discussed results, and revised the manuscript. All authors contributed to the article and approved the submitted version.

## Supplementary material

Supplementary Figure 1 Correlation coefficients for flag leaf length (FLL) **(A)**, flag leaf width (FLW) **(B)**, and flag leaf area (FLA) **(C)** of parents and the WC12 population in different environments. *** and **** represent significant at *P* < 0.005 and *P* < 0.001, respectively

Supplementary Figure 2 Conditional quantitative trait loci (QTL) analysis among flag leaf-related traits. Flag leaf area (FLA) trait on flag leaf length (FLL) and flag leaf width (FLW) traits on chromosomes 2B **(A)** and 2D **(B)**

Supplementary Figure 3 Additive effects of two flag leaf length (FLL)-related QTL **(A)**, three flag leaf width (FLW)-related QTL **(B)**, and two flag leaf area (FLA)-related QTL **(C)** on FLL, FLW, and FLA, respectively. + and − represent lines with and without the positive alleles of the target QTL based on the flanking markers of the corresponding QTL, respectively. **** represents significant at *P* < 0.001

Supplementary Figure 4 Expression pattern of genes within the *QFll/Fla.cib-2B* interval. **(A–D)** represent leaf, grain, spike, and root by Triticeae Multi-omics Center, respectively; **(E–H)** represent leaves/shoots, grain, spike, and roots by Wheat Expression Browser, respectively.

Supplementary Figure 5 QTL analysis **(A)**, genetic map **(B)**, and physical map **(C)** of *QFlw.cib-4B.3*

Supplementary Figure 6 QTL analysis **(A)**, genetic map **(B)**, and physical map **(C)** of *QFll/Flw/Fla.cib-2D*

Supplementary Figure 7 QTL analysis **(A)**, genetic map **(B)**, and physical map **(C)** of *QFll/Fla.cib-2B*

Supplementary Figure 8 Effect analysis of *QFll/Fla.cib-2B* **(A)**, *QFlw.cib-4B.3* **(B)**, and *QFll/Flw/Fla.cib-2D* **(C)** on grain yield per plant (GYP) in WC12 population. W7268 and CY12 represent lines with alleles from W7268 and CY12, respectively; *, **, ***, and **** represent significant at *P* < 0.05, *P* < 0.01, *P* < 0.005, and *P* < 0.001, respectively.
