# Supplementary material for: Genetic dissection of quantitative trait loci for flag leaf size in bread wheat (Triticum aestivum L.)
Source: Front Plant Sci. 2022 Dec 14;13:1047899. doi: 10.3389/fpls.2022.1047899 (PMC9807109; doi:10.3389/fpls.2022.1047899)
Supplement: Supplementary Figure 1 — Correlation coefficients for flag leaf length (FLL) (A), flag leaf width (FLW) (B), and flag leaf area (FLA) (C) of parents and the WC12 population in different environments. *** and **** represent significant at P < 0.005 and P < 0.001, respectively [file Presentation_1.pptx]

## Slide 1
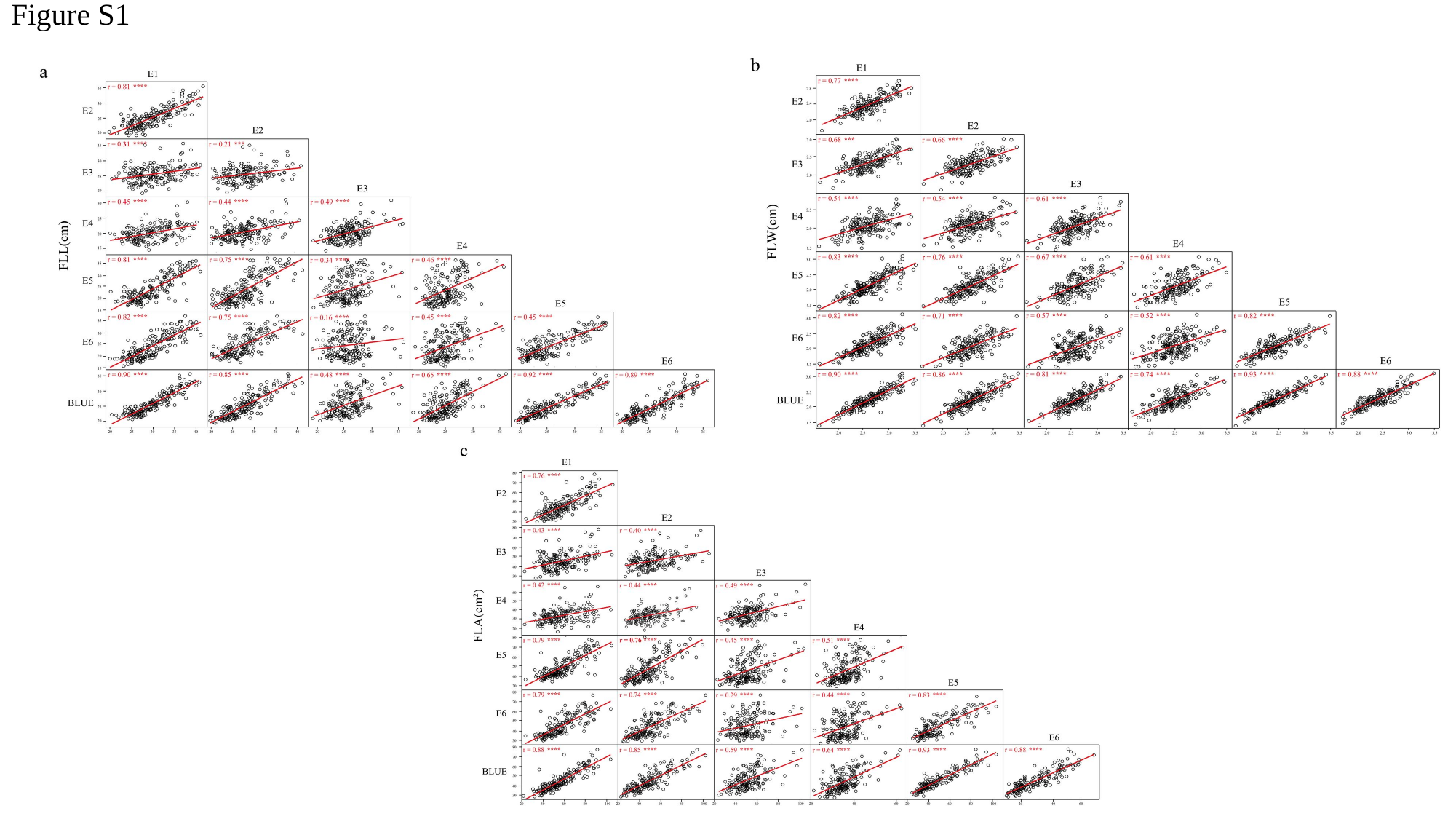

# Figure S1

## Slide 2
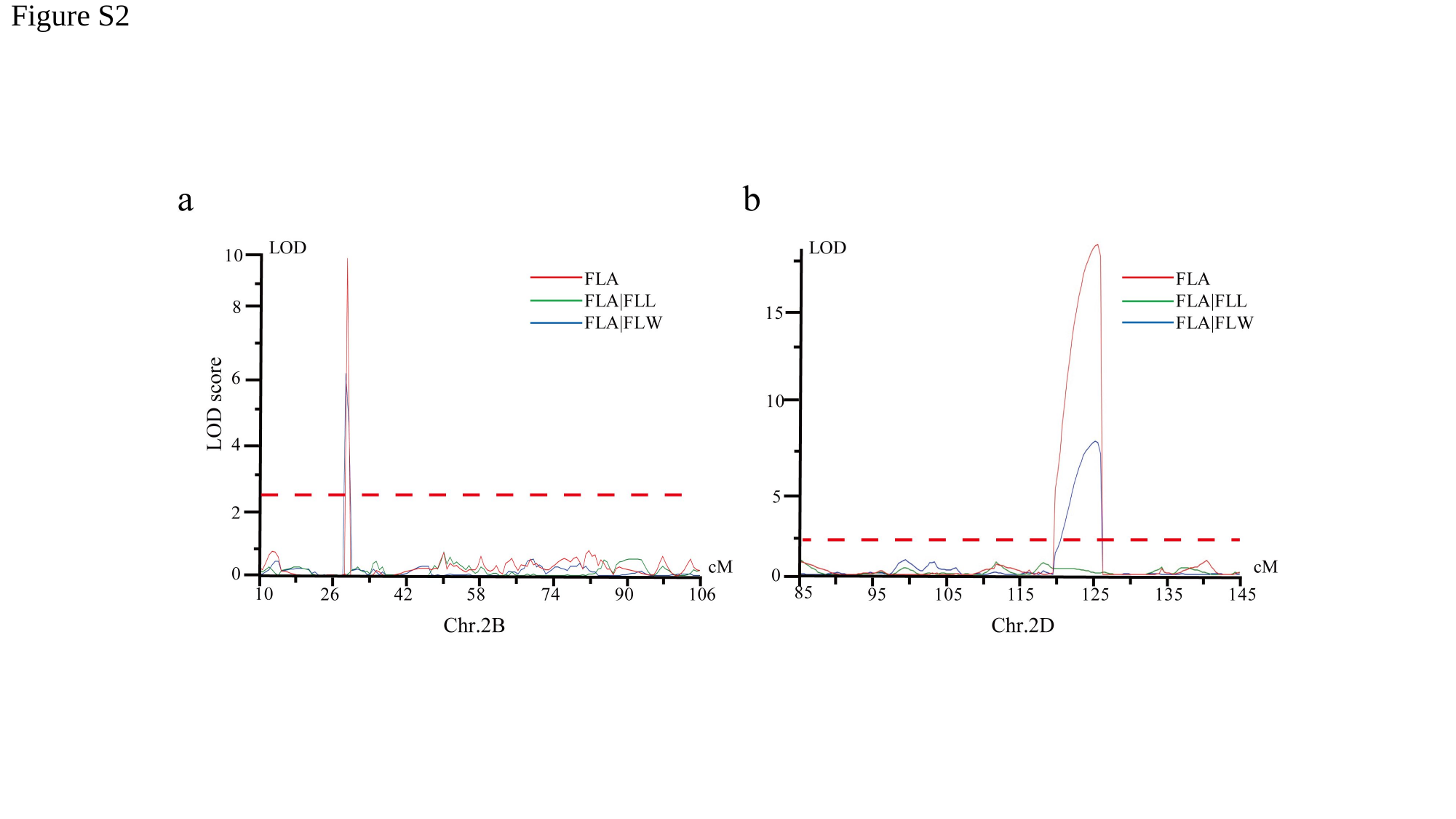

# Figure S2

## Slide 3
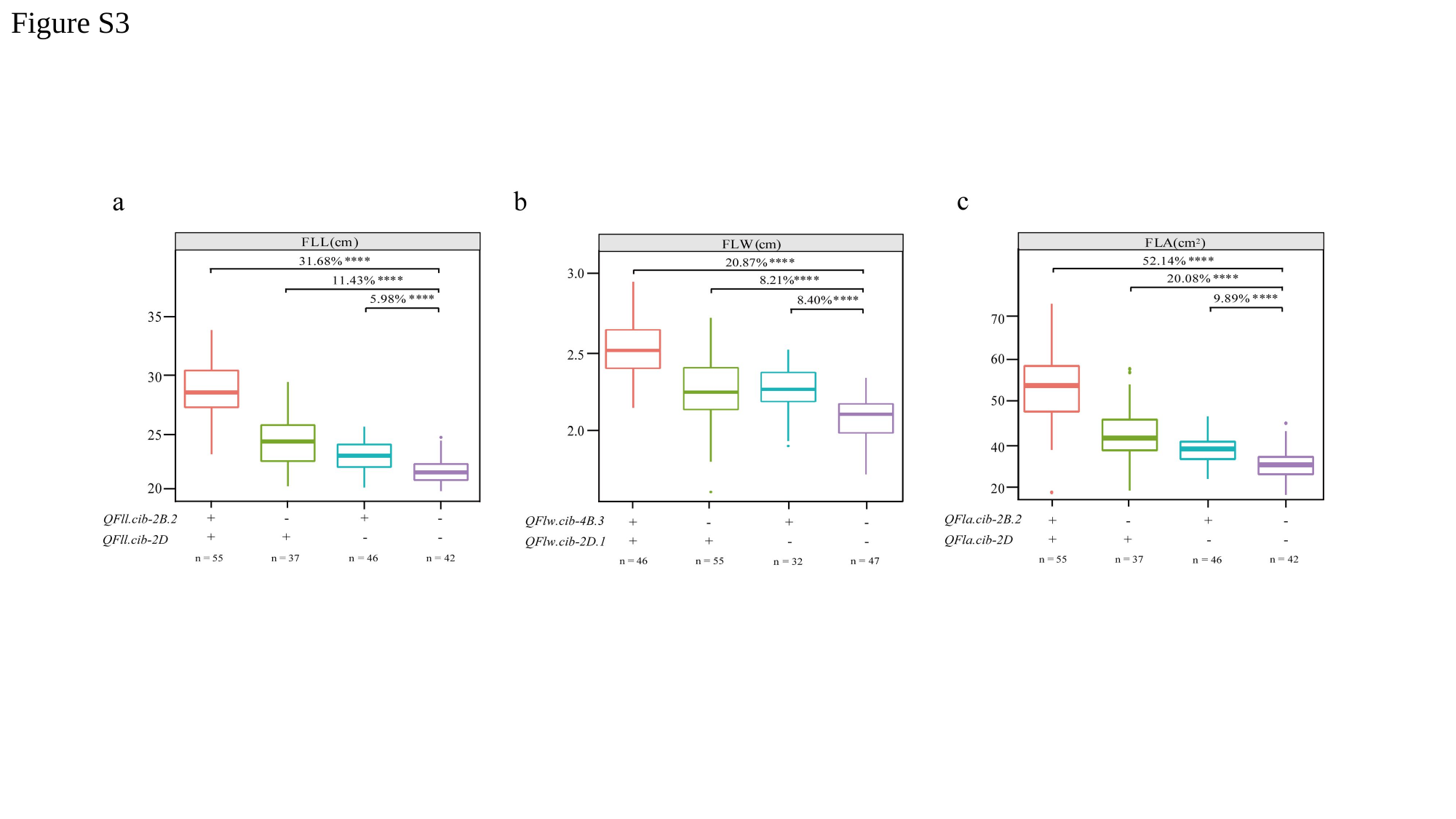

# Figure S3

## Slide 4
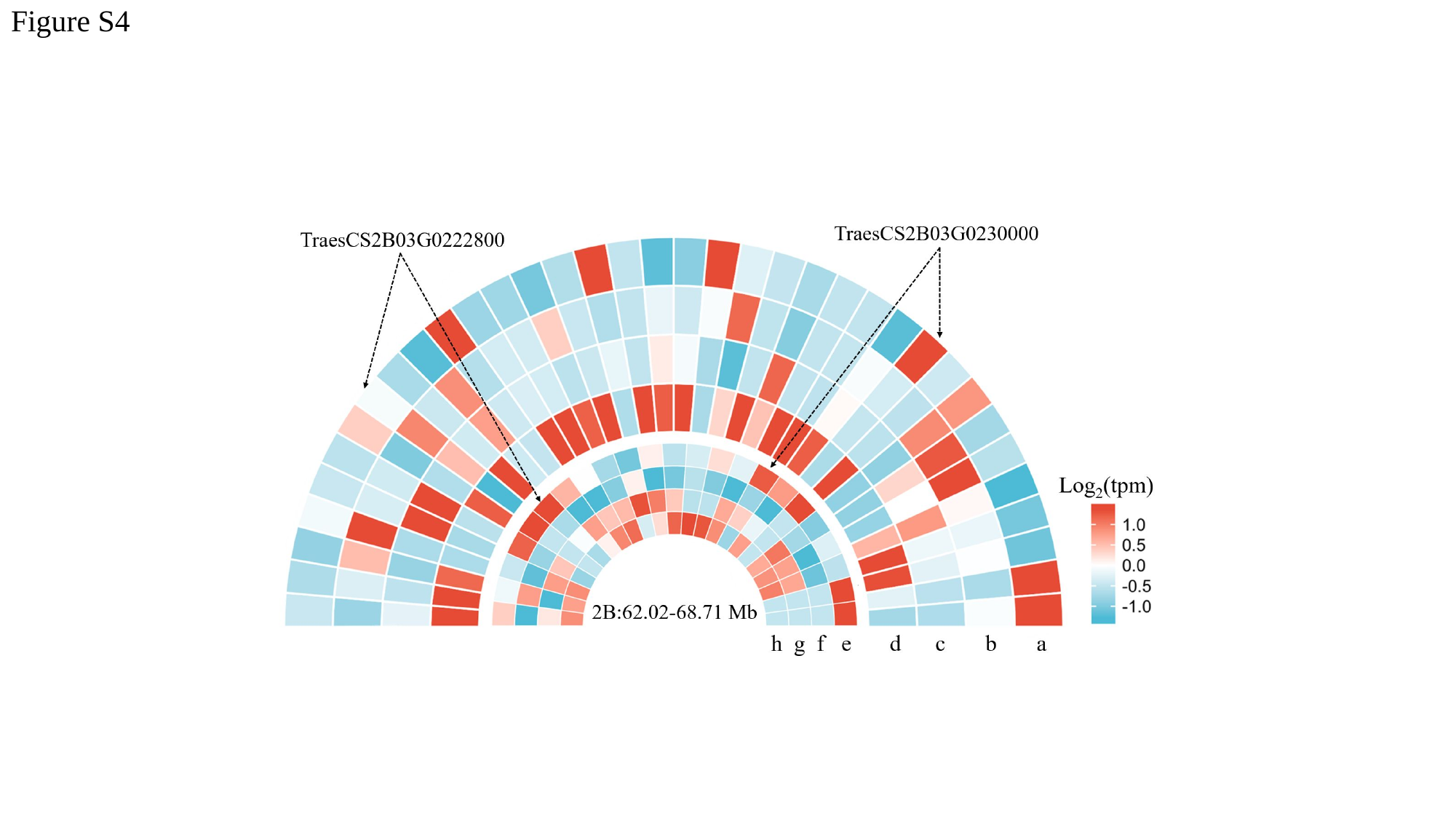

# Figure S4

## Slide 5
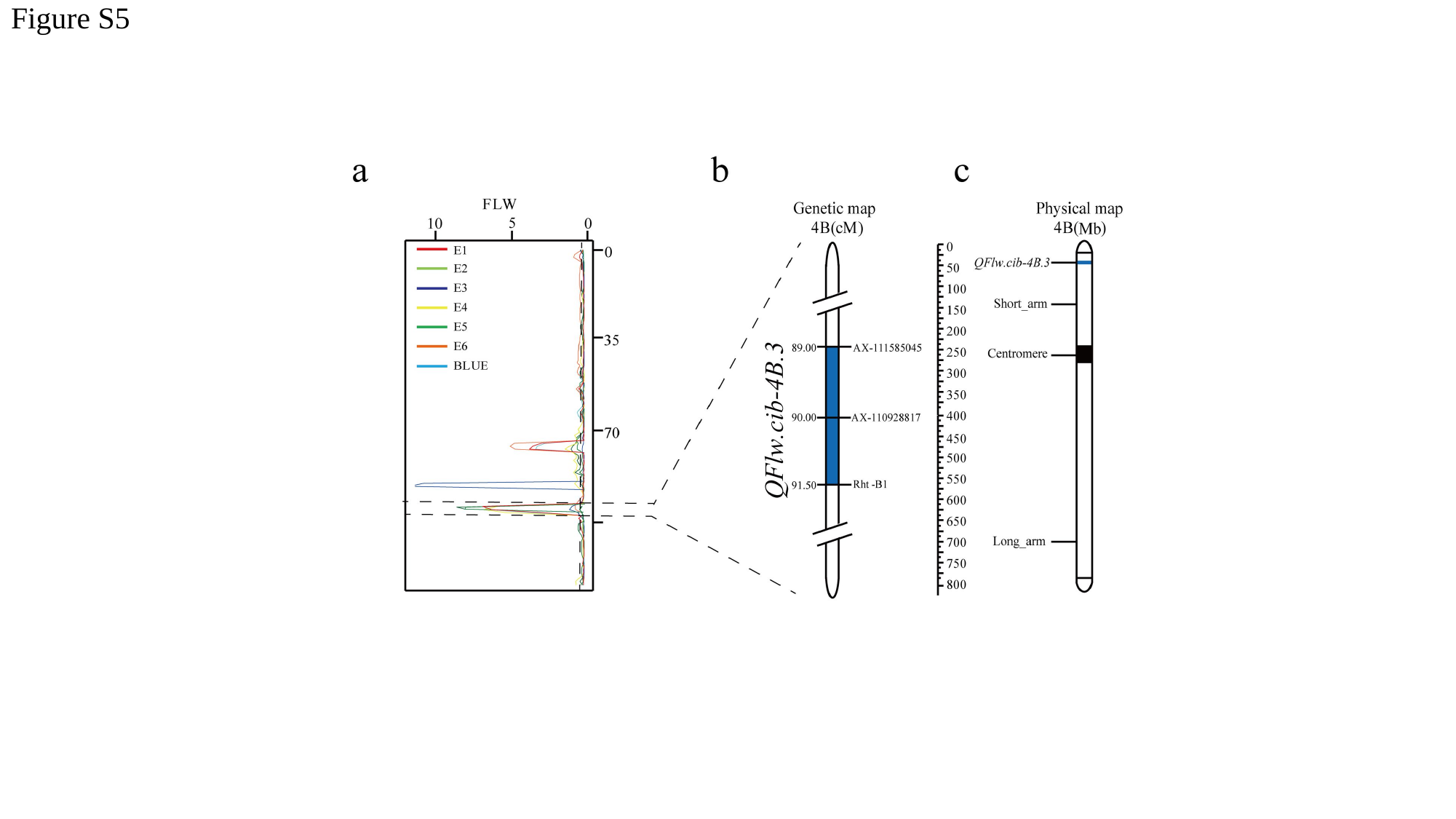

# Figure S5

## Slide 6
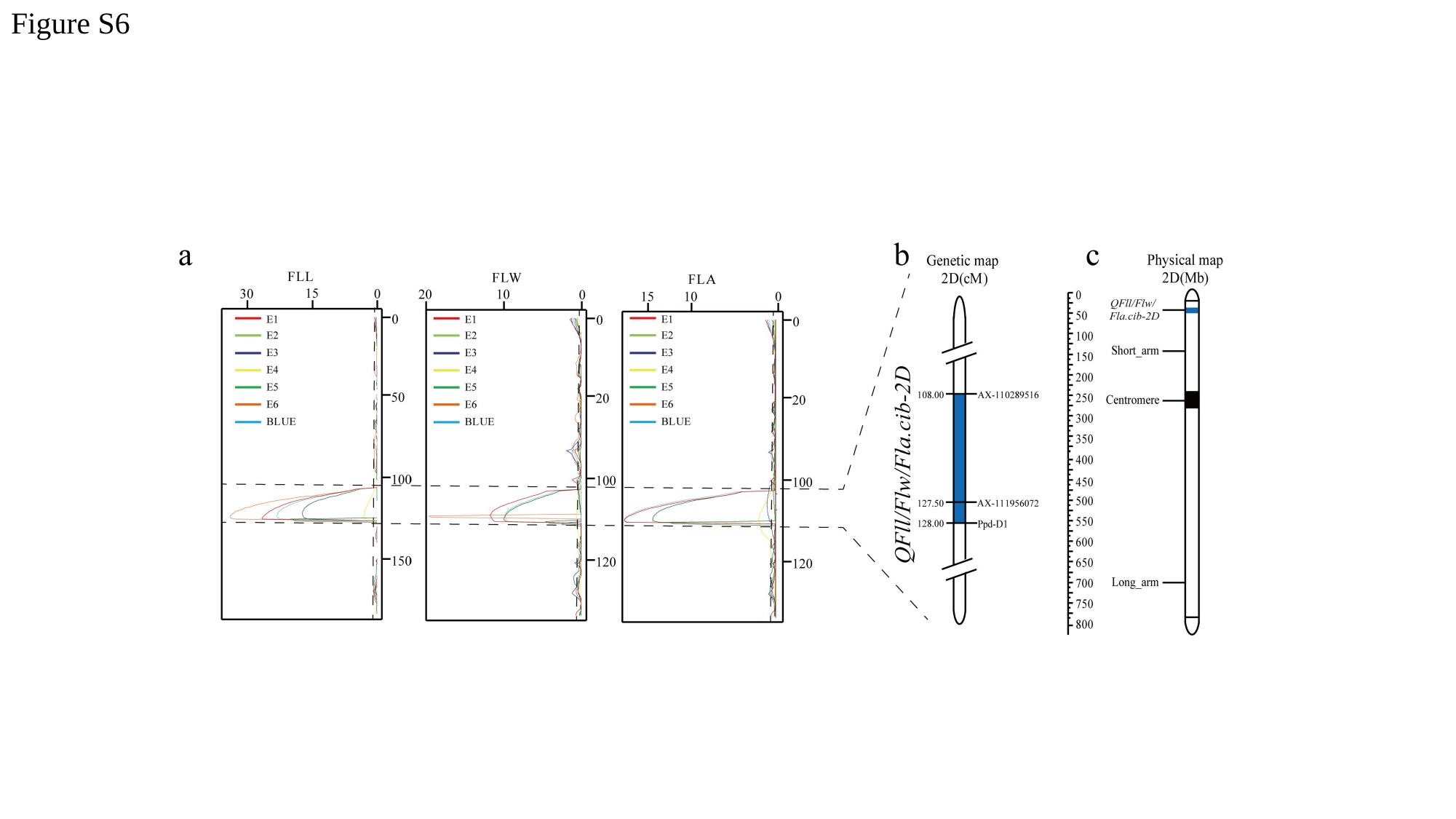

# Figure S6

## Slide 7
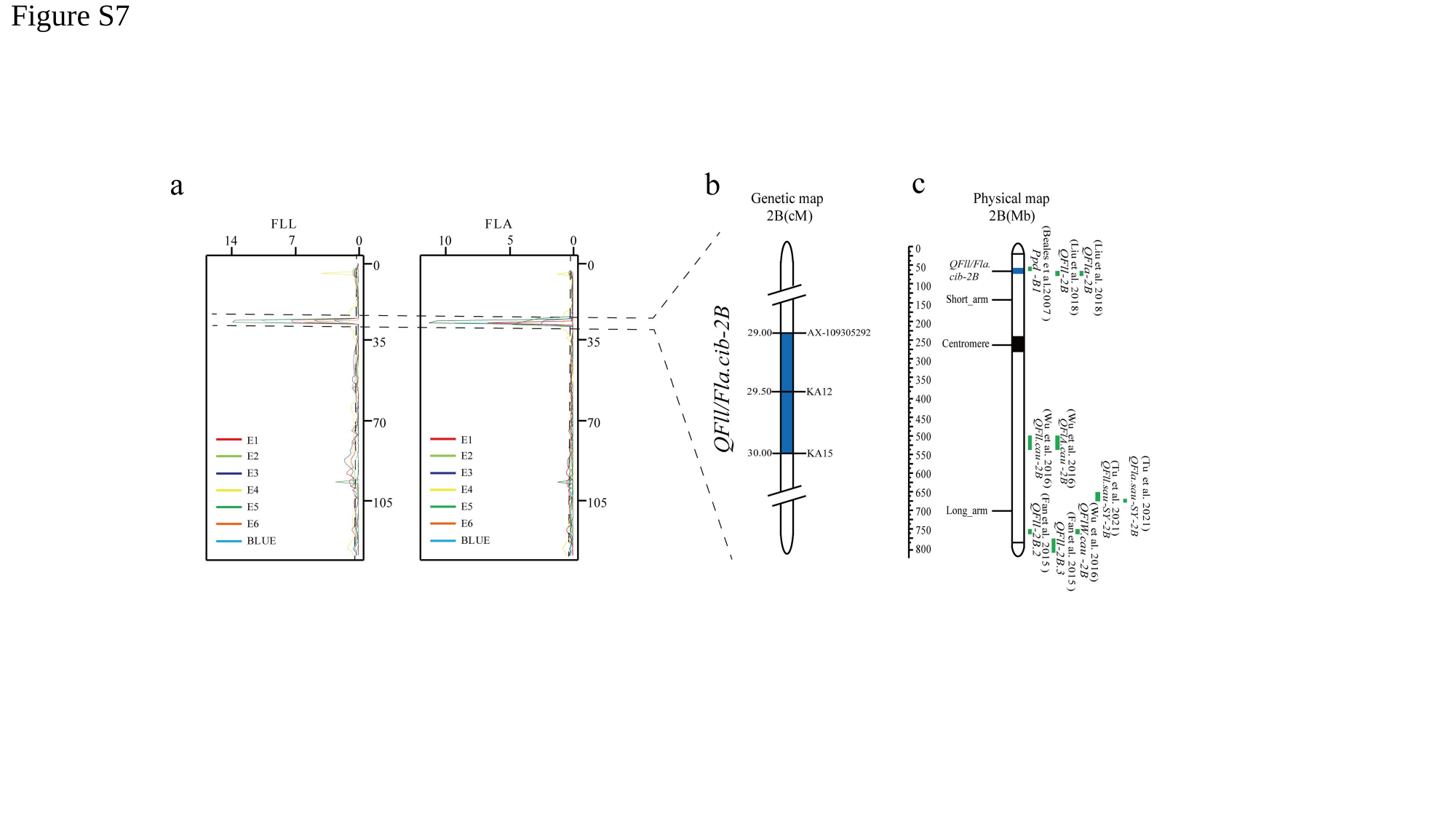

# Figure S7

## Slide 8
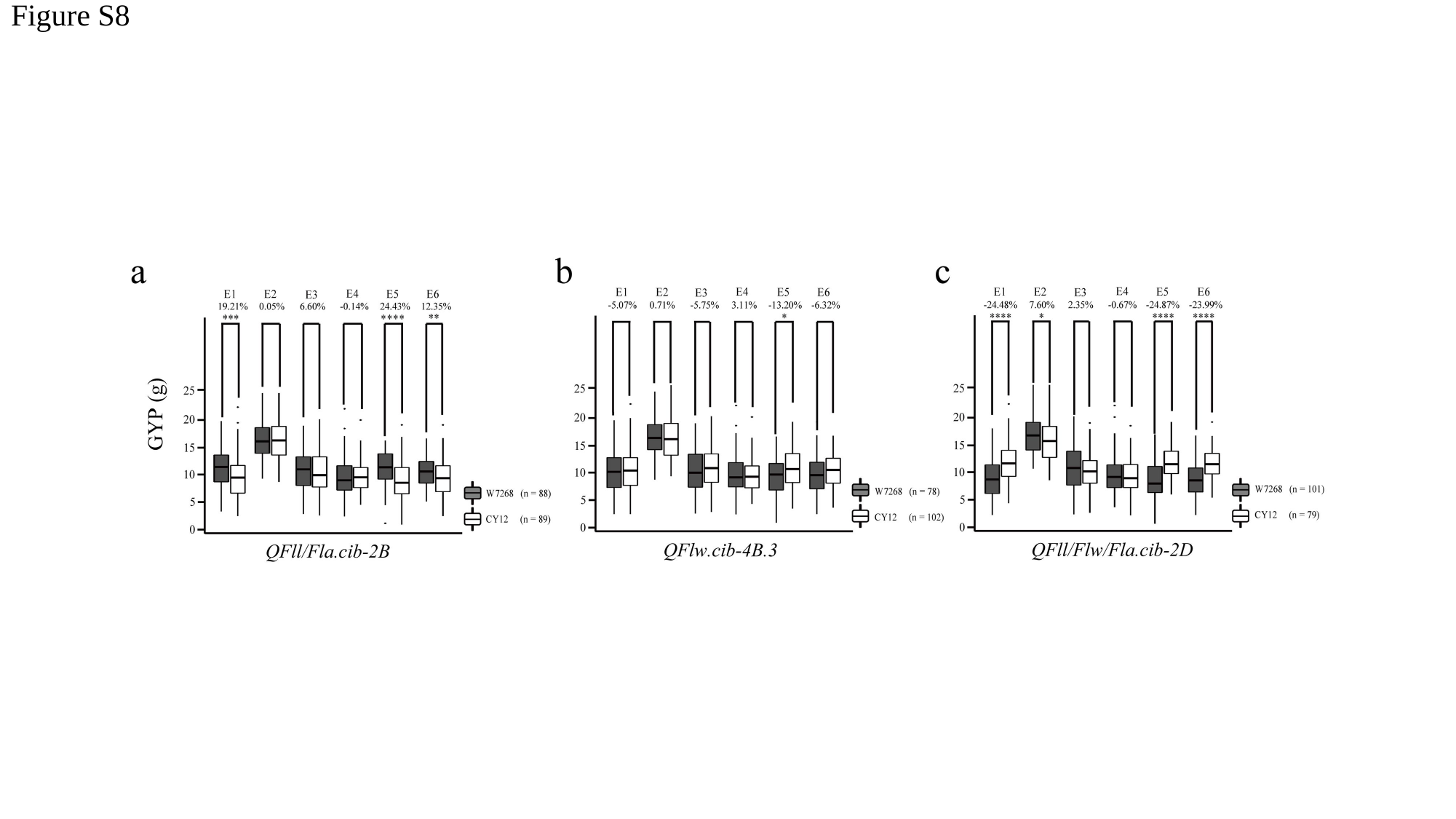

# Figure S8
